# UGT1A4*3 polymorphism influences serum concentration and therapeutic effect of lamotrigine for epilepsy treatment: A meta-analysis

**DOI:** 10.1371/journal.pone.0307377

**Published:** 2024-07-18

**Authors:** Zhimei Jiang, Yuzhi Fu, Hongxin Shen

**Affiliations:** 1 Department of Pharmacy, West China Second University Hospital of Sichuan University, Chengdu, China; 2 Evidence-Based Pharmacy Center, West China Second University Hospital of Sichuan University, Chengdu, China; 3 Key Laboratory of Birth Defects and Related Diseases of Women and Children, Ministry of Education, Sichuan University, Chengdu, China; Makerere University, UGANDA

## Abstract

**Background:**

Lamotrigine as a broad-spectrum antiepileptic drug, is widely applied and its clinical efficacy is highly recognized. However, significant differences are observed in blood drug concentration of lamotrigine among individuals, which may have an impact on its efficacy. UGT1A4 is the main metabolic enzyme. However, it was inconsistent for the influence of UGT1A4 genetic polymorphism on concentration and efficacy of lamotrigine therapy. This study aimed to evaluate the influences of UGT1A4*3 genetic polymorphisms on lamotrigine concentration and therapeutic effect through meta-analysis.

**Methods:**

The literature search was conducted in Medline, Embase, PubMed, Web of Science, Wan Fang Database, China National Knowledge Infrastructure, China Science and Technology Journal Database until January 2024. The primary outcome included the mean serum concentration, concentration-to-dose-ratio by body weight (CDR), or efficacy related to different UGT1A4*3 genotype for lamotrigine therapy. Data were collected to access the Mean Difference or odds ratio with 95% confidence interval. Meta-analysis was performed by RevMan 5.2.

**Results:**

A total of eleven studies were enrolled. The meta-analysis for mean serum concentration of lamotrigine showed no significant difference between patients carrying TT genotypes and TG and GG genotypes group (MD: 0.12, 95% [-0.35, 0.58], *P* = 0.62). There was significant difference in CDR (MD: 0.49, 95% [0.03, 0.94], *P* = 0.04) and therapeutic efficacy (OR: 7.18, 95% [4.01, 12.83], *P*<0.00001) of lamotrigine, however no significant difference was found in subgroup analysis of CDR of children (MD: 0.03, 95% [-0.35, 0.42], P = 0.87) between patients carrying TT genotypes and TG and GG genotypes group.

**Conclusions:**

Polymorphism of UGT1A4*3 influenced the CDR and therapeutic efficacy of lamotrigine for antiepileptic therapy. Genotype analysis provided reference for personalized medication in the future. However, more high-quality evidences are necessary for precise and definitive conclusion.

## Introduction

Epilepsy is one of the most prevalent brain conditions in the world, it nearly affects over 70 million of people [1]. It is a disease complex with multiple causes and strong genetic predisposition. Identification of causes has important therapeutic significance. The categories of causes are structural, metabolic, genetic, immune, infectious, and unknown [2]. The characteristic of epileptic seizures is stereotyped behavioral alterations. Therefore, epilepsy is mostly diagnosed through detailed clinical history and observation of a seizure by reliable eyewitness. Surgery is an effective option for long-term seizure freedom. Antiseizure medications are the major choices for most people with epilepsy. Its aim is to stop seizures at the earliest moment which may reduce morbidity and the risk of premature death especially related with convulsions [3, 4]. Over 25 medications are available worldwide for epilepsy treatment. However, it is effective for current drugs in only about 66% patients in high-income countries [5]. Lamotrigine (LTG) is a novel antiepileptic drug and a mood stabilizer. It mainly exerts antiepileptic effects by blocking presynaptic membrane voltage sensitive sodium ion channels, and inhibiting the release of glutamate and aspartic acid. It is necessary to start with a small dose and gradually increase the dosage [6]. However, the clinical efficacy of LTG varies greatly among individuals, the same for the occurrence of adverse reactions such as rash, nausea, vomiting, diarrhea, and headache. Taking the same dose of LTG may result in significant individual differences. For example, some patients are ineffective and others potentially experience adverse reactions. This is related to the significant individual differences in LTG steady-state blood drug valley concentration [7]. Furthermore, plasma clearance of LTG could be increased by hepatic enzyme induced drugs (such as phenytoin sodium, carbamazepine, oxcarbazepine) through inducing hepatic glucuronic acid binding reactions [8]. Valproate sodium reduces the plasma clearance rate of LTG through inhibiting the reactions [9]. Therefore, application of therapeutic drug monitoring (TDM) is clinically needed to realize personalized treatment with LTG [10]. Accordingly, TDM of LTG was considered as “recommended” and therapeutic window was suggested as 3–14 μg/mL for concentrations of LTG by the TDM expert group of the Arbeitsgemeinschaft für Neuropsychopharmakologie und Pharmakopsychiatrie consensus and ILAE Commission on Therapeutic Strategies [11, 12]. At present, LTG dose-escalation regimens are mostly used by clinical physicians. Then the dosage regimens are adjusted empirically or based on TDM results which are time-consuming. Therefore, how to control epilepsy quickly and effectively, and enable LTG to reach therapeutic window is an urgent problem that needs to be solved in clinical practice.

Researches found that genetic factors such as mutations in metabolic enzymes and transporters might be one of the important reasons for the large individual differences in LTG serum concentration [13, 14]. It is also a hot research topic in recent years. With the deepening research of pharmacogenomics, the impact of polymorphisms in drug metabolism enzymes and transporter on the serum concentration of LTG is increasingly being valued. LTG is mainly metabolized by UDP glycuronosyltransferase (UGT). It is metabolized into inactive glucuronic acid binding products, and then excreted from the body through the kidneys. UGT1A4 is the main metabolic enzyme [15]. In its coding region, UGT1A4*3 is the most common single nucleotide gene polymorphism (SNP) [16]. Zhou *et al*. [16] found that mutations in UGT1A4 70C>A and 142T>G could lead to a decrease in UGT enzyme activity through in vitro studies, resulting in a decrease in LTG clearance rate. A study was conducted by Chang et al. [17] on the correlation between LTG blood drug concentration and UGT1A4 70C>A and 142T>G genotypes in 106 Chinese epilepsy patients. It was found that the LTG standardized blood drug concentration/dose/normalized by body weight (CDR) of UGT1A4 142TT wild-type carriers was significantly higher than that of TG+GG carriers. However, some researches did not find the UGT1A4 142T>G gene mutation could affect the blood drug concentration of LTG. It is obviously that the conclusions about the influence of UGT1A4*3 polymorphism on the concentration and therapeutic efficacy of LTG is not entirely consistent. Furthermore, there is no systematic review discussing the collaborative impact by UGT1A4*3 polymorphism between serum concentration and therapeutic efficacy of LTG.

Therefore, our study systematically evaluated the effects of UGT1A4*3 polymorphisms on LTG serum concentration and therapeutic efficacy through meta-analysis. It was meaningful to provide a underlying mechanism of interindividual variation in the LTG treatment. The influence was useful to be considered as a factor when pharmacokinetic model was determined for LTG dose adjustment.

## Materials and methods

### Eligibility criteria

The present review included cohort researches investigating serum concentrations and effect of LTG influenced by UGT1A4*3 genetic polymorphisms during epilepsy. Types of prospective and retrospective both were included. The intervention was treated with LTG for epilepsy monotherapy. The exposed group showed TT genotype, whereas the control group was TG and GG genotype of UGT1A4*3. Eligible researches reported one of the following outcomes: mean serum concentration, CDR, or efficacy related to different UGT1A4*3 genotype. Studies published in English or Chinese were considered.

### Literature search

Four English language databases including Medline, Embase, PubMed, Web of Science were searched. Three Chinese databases including Wan Fang Database, China National Knowledge Infrastructure, China Science and Technology Journal Database were also searched.

The following principal search terms were used: “lamotrigine” and “gene” or “genetic” or “polymorphisms” and “UGT1A4”. They were combined to search for relevant studies. Furthermore, it was restricted to human studies. We would check additional studies in reference lists of included studies, and contact with authors to request missing data. The last retrieval was performed on January, 2024.

### Study selection and data extraction

Two trained reviewers independently screened potentially eligible studies based on title and abstract, and then read full texts for final eligibility. Disagreements were resolved by a third person. The information derived from included studies was collected and shown in a table including the following data: general characteristics of studies and patients, interventions and comparisons, outcomes (mean serum concentration, CDR, efficacy), assess of Hardy–Weinberg equilibrium. The MSC referred to the average steady-state valley concentration of LTG, and the CDR was derived through the formula: LTG steady-state valley plasma concentration/ LTG dose / weight. The efficacy of LTG was graded using Engel method [18]. It was determined by monitoring the frequency of epileptic seizures in patients during a one-year follow-up.

### Quality assessment

The quality of included studies was evaluated according to Newcastle-Ottawa Scale (NOS). It includes three items. Selection of case and controls: 1) is the case definition adequate (Yes, with independent validation, score a star)? 2) Representativeness of the cases (consecutive or obviously representative series of cases, score a star); 3) Selection of controls (community controls, score a star); 4) Definition of controls (no history of disease (endpoint), score a star); Comparability of cases and controls: 1) Comparability of cases and controls on the basis of the design or analysis (study controls for (select the most important factor), score a star; study controls for any additional factor, score a star); Exposure: 1)Ascertainment of exposure (secure record, score a star; structured interview where blind to case/control status, score a star); 2)Same method of ascertainment for cases and controls (yes, score a star); 3)Non-response rate (same rate for both groups, score a star). It was not suitable to assess publication bias because of insufficient number of included studies.

### Statistical analysis

Statistical analyses were performed with Review Manager Version 5.2 (Copenhagen: The Nordic Cochrane Centre, 2012). It could perform meta-analysis of the data entered, and present the results graphically. The data of mean serum concentration and CDR outcomes were continuous data which were analyzed for Mean Difference (MD). The data of efficacy outcome was dichotomous data which were analyzed for odds ratio (OR) with 95% confidence interval (CI). Due to the fact that all included studies might not be highly consistent, i.e. there might be differences between subjects and interventions, and the resulting effect size might not be a fixed value, a random effects model was chosen for meta-analysis. Study heterogeneity was determined via statistical analyses, using the Q statistic for homogeneity and the I-squared (*I*^*2*^) statistic. A P value of < 0.10 or *I*^*2*^ >50% was considered to indicate significant heterogeneity. When only minimum (Min) and maximum (Max) were available for a specific sample, standard deviation (SD) was estimated (SD = (Max-Min)/4) [19]. When Mean and SD in two groups were merged, formulas were as follows: mean (M) = (N_1_M_1_+N_2_M_2_)/(N_1_+N_2_), $SD=\sqrt{\frac{{(N1-1)SD1}^{2}+{(N2-1)SD2}^{2}+\frac{N1N2}{N1+N2}({M1}^{2+}{M2}^{2}-2M1M2)}{N1+N2–1}}$ (group A sample size N_1_, mean M_1_, standard deviation SD_1_; group B sample sizeN2, mean M_2_, standard deviation SD_2_) [20]. Sensitivity analysis was performed by individually excluding each study to assess the quality and consistency of the results.

## Results

### Search results and characteristics of enrolled studies

**Fig 1:**
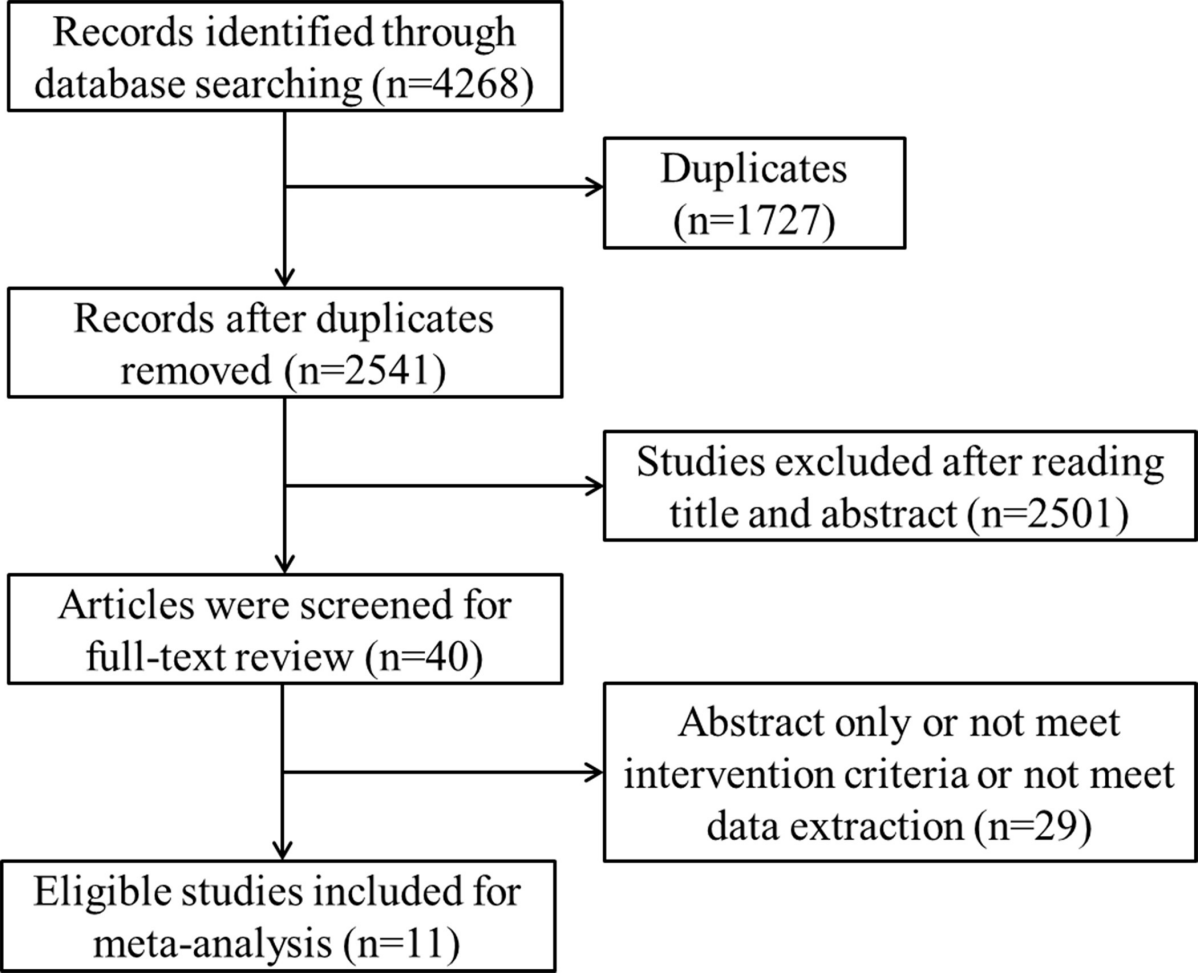
Follow diagram of selecting study.

A total of 4268 records were identified by initial database search. After first screening of the title and abstract, 4219 articles were excluded. Following the full-text review, 11 studies were included (Fig 1). Of the 11 eligible studies, 7 studies were conducted for mean serum concentration for 541 patients; 8 studies enrolled 680 patients with the determination of CDR outcome; 3 studies shown efficacy outcome for 308 patients. General characteristic of included studies were shown in Table 1. Detailed NOS results of quality assessment of included studies were shown in Table 2.

**Table 1:** Characteristics of included studies.

Author(year)	Datasource/country	Number of patients (males)	Age, y	Outcome measurement of LTG	Administration dosage	Hardy–Weinberg equilibrium
Suzuki 2019 [21]	Japanese	103(Unclear)	42.2±16.2	mean serum concentration	100mg/d	Unclear
Petrenaite 2022 [22]	Danish	195(87)	18–67	mean serum concentration	TT:335±168mg/d	Yes
					TG:320±180mg/d	
Gulcebi 2011 [23]	Turkish	35(10)	18–50	mean serum concentration	3.07±0.15mg/kg/d	Unclear
Zhou 2015 [24]	China	119(57)	29±12	mean serum concentration; CDR	TT2.22:(1.46–3.12)mg/kg	Yes
					GT+TT:2.27(1.96–3.08)mg/kg	
Liu 2015 [25]	China	56(31)	TT:7.74±2.53	mean serum concentration; CDR	TT:68.82±36.81mg/d	Yes
					TG+GG:46.02±12.43mg/d	
			TG+GG:8.55±3.14			
Lou 2021 [26]	China	49(22)	8.0(6.0–12.0)	mean serum concentration; CDR	100mg/d	Yes
Liu 2014 [27]	China	160(98)	8.25±3.18	mean serum concentration; CDR	2.76±1.65mg/kg	Unclear
Yang 2013 [28]	China	103(40)	32.07±12.88	CDR; efficacy	0.41–4.30mg/Kg	Yes
Chang 2014 [17]	China	106(40)	44.55±11.79	CDR; efficacy	0.41–4.30 mg/kg	Yes
Du 2016[29]	China	102(57)	12.33±6.13	CDR; efficacy	2.74±1.17 mg/kg	Unclear
Reimers 2016 [29]	Norway	127(Unclear)	12–65	CDR	278.3(Mean)	Unclear

**Table 2:** Quality assessment of included studies using NOS.

Included studies	Selection of case and controls				Comparability of cases and controls	Exposure		
	is the case definition adequate	Representativeness of the cases	Selection of controls	Definition of controls	Comparability of cases and controls on the basis of the design or analysis	Ascertainment of exposure	Same method of ascertainment for cases and controls	Non-response rate
Suzuki 2019 [21]	★	-	-	★	★	★★	★	-
Petrenaite 2022 [22]	★	-	-	★	★★	★★	★	-
Gulcebi 2011 [23]	★	-	-	★	★	★★	★	-
Zhou 2015 [24]	★	-	-	★	★★	★★	★	-
Liu 2015 [25]	★	-	-	★	★★	★★	★	-
Lou 2021 [26]	★	-	-	★	★★	★★	★	-
Liu 2014 [27]	★	-	-	★	★	★★	★	-
Yang 2013 [28]	★	-	-	★	★★	★★	★	-
Chang 2014 [17]	★	-	-	★	★★	★★	★	-
Du 2016 [29]	★	-	-	★	★★	★★	★	-
Reimers 2016 [30]	★	-	-	★	★★	★★	★	-

### Effect of UGT1A4*3 polymorphism on LTG mean serum concentration

**Fig 2:**
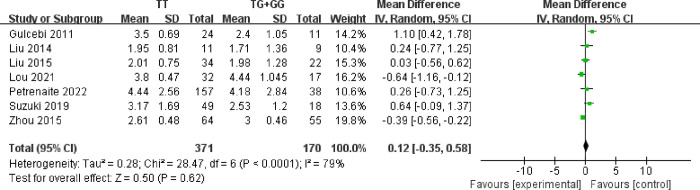
Forest plot of UGT1A4*3 polymorphism on mean serum concentration of LTG.

Seven studies [21–27] involving 360 patients in the TT genotype group and 161 patients in the TG and GG genotype group reported mean serum concentration of LTG based on the UGT1A4*3 polymorphism during LTG monotherapy (Fig 2). Random-effects model was chosen because of statistical heterogeneity (*I*^*2*^ = 82%). Pooled analysis of data showed no significant difference between the LTG serum concentration of subjects carrying TT genotype with TG and GG genotype (MD: 0.12, 95% [-0.35, 0.58], *P* = 0.62).

### Effect of UGT1A4*3 polymorphism on CDR

**Fig 3:**
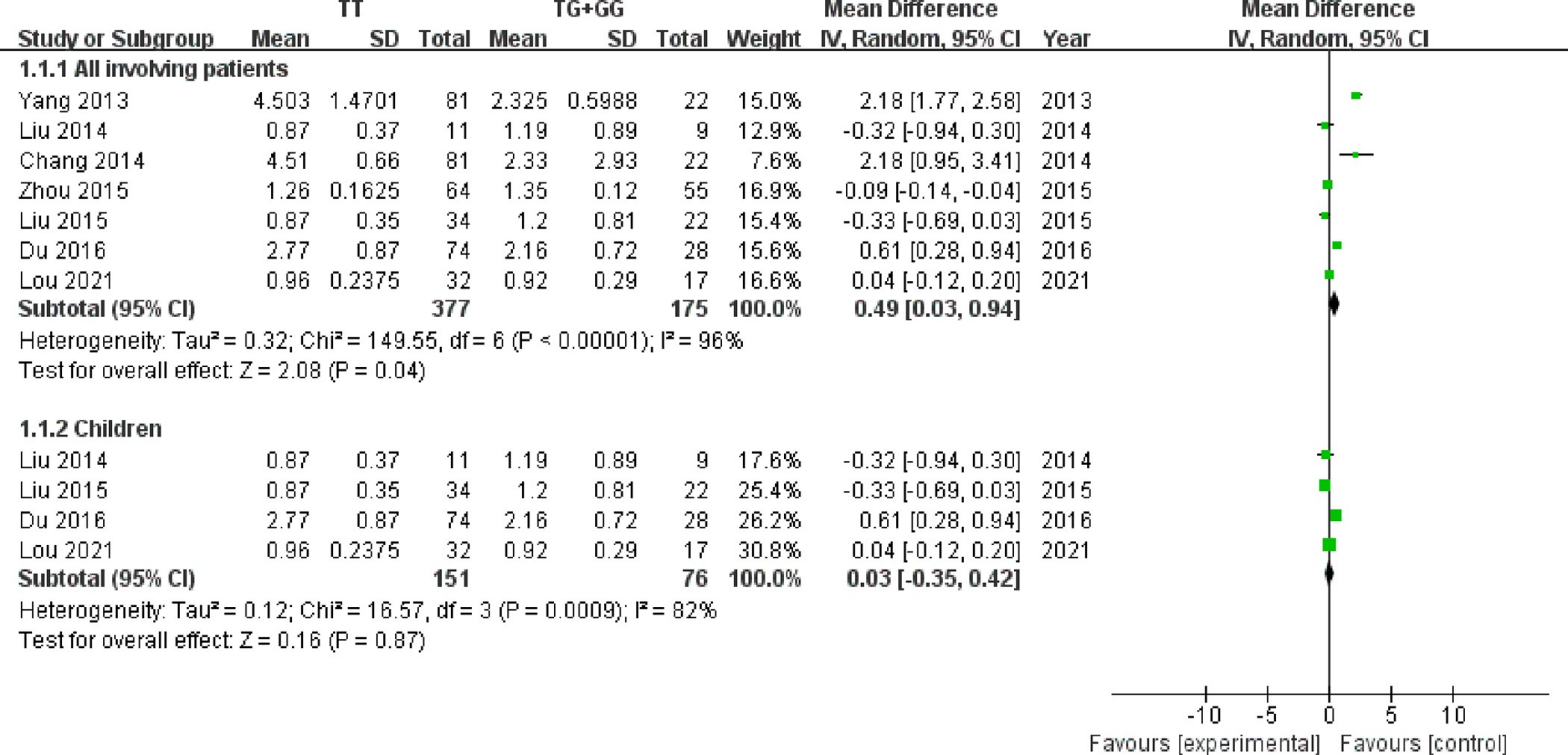
Forest plot of UGT1A4*3 polymorphism on CDR of LTG.

A total of 8 studies were included to pooled analysis CDR difference of LTG based on UGT1A4*3 polymorphism. 7 records [17, 24–29] reported appropriate data types for Meta-analysis (Fig 3). Random-effects model was used (*I* ^*2*^ = 96%). Based on the data from those studies, patients with TT genotype yielded a significantly higher CDR of LTG than with TG and GG genotype (MD: 0.49, 95% [0.03, 0.94], *P* = 0.04). One study [30] only described individuals heterozygous for UGT1A4*3 had a significantly lower CDR than individuals with wild-type (TT) without detailed data. Clearly, it was consistent with our systematic Meta-analysis results. However, through subgroup analysis of CDR in children, there was no significant difference between patients carrying TT genotype group and TG and GG genotype group (MD: 0.03, 95% [-0.35, 0.42], P = 0.87).

### Effect of UGT1A4*3 polymorphism on therapeutic efficacy of LTG

**Fig 4:**
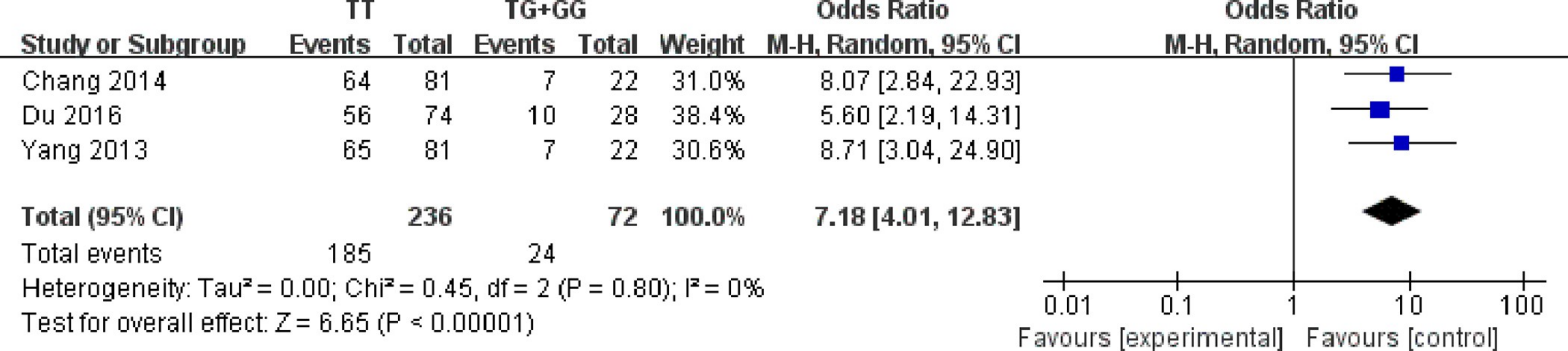
Forest plot of UGT1A4*3 polymorphism on therapeutic effect of LTG.

Only three studies [28, 29], involving 308 patients, reported the therapeutic efficacy of LTG based on UGT1A4*3 polymorphism. No statistical heterogeneity existed among the article results (*I* ^*2*^ = 0%). Random-effects model was used to perform the systematic analysis. There was significant difference between individuals with TT genotype and the control group in the therapeutic effect of LTG (OR: 7.18, 95% [4.01, 12.83], *P*<0.00001) (Fig 4). The results indicated that the therapeutic effect of LTG with TT genotype was better than that with TG/GG.

## Discussion

As reported, the therapeutic response of LTG therapy relied on its serum concentration. Unfortunately, there were significant interindividual differences in serum concentration or CDR among subjects [31]. It was widely known that coadministration of valproate significantly increased LTG concentrations [32, 33]. Therefore, we focused on the influence of UGT1A4 genetic polymorphisms based on LTG monotherpy or simultaneous medication of non-interacting antiepileptic drugs. UGT1A4 was the main metabolic enzymes of LTG into inactive glucuronic acid conjugates [24]. Meanwhile, it was controversial for the correlation between genotype of UGT1A4*3 and serum or efficacy of LTG. This study systematically evaluated the influence of UGT1A4*3 on serum concentration and efficacy of LTG therapy through meta-analysis.

Mean serum and CDR were the most common parameters in eligible studies. It was interesting that there was no remarkable difference in mean serum concentration of LTG between patients carrying TT genotypes group and TG and GG genotypes group. However, significant difference was observed in CDR of LTG between patients carrying TT genotypes group and TG and GG genotypes group. Namely, the CDR level of LTG for patients carrying TT genotype was higher than that for patients carrying TG and GG genotype. Due to individual differences in weight and drug dosage among patients, CDR was adjusted based on daily dose and individual weight. It is standardized blood drug concentrations which excludes the influence of body weight and dosage on blood drug concentration. Therefore, it suggested that CDR parameter was to be a preferred detection indicator, to provide reference for personalized medication and achieve rational administration in different genotype of UGT1A4*3 patients. Through subgroup analysis of CDR in children, the meta-analysis suggested that there was no significant difference. It might be attributed to low activity of glucuronic acid transferase in young children [34]. Therefore, adjusting dosage based on the overall population genetic polymorphism could not guarantee the safety of medication in children. It is necessary to consider organ maturation and individual development of drug metabolism enzymes in order to provide reasonable dosage.

Meta-analysis was performed on therapeutic efficacy of LTG in patients with different genotype of UGT1A4*3. It showed that the clinical efficacy of LTG therapy for TT genotype patients was better than that for TG and GG genotype patients. Reasonably, it was consistent with the CDR outcome results. Therefore, the conclusions may be helpful to understand the individual differences of LTG therapy. Patient’s genotype can be considered as a factor to establish pharmacokinetic model for reasonable dosage of LTG. On the other hand, its pharmacokinetic differences may be influenced by combined effects of multiple genes, and hepatic enzyme induced and inhibited drugs. Other significant factors are necessary to be further investigation for LTG individualized medication.

The present study still has limitations. First, because of the varied sample sizes of enrolled studies, inconsistency in the methods applied to determine LTG concentration and different dosage, the results might be biased due to clinical heterogeneity. The quality of the included studies was categorized as having a medium risk of bias (awarded six or seven stars). Five studies did not describe assess of Hardy-Weinberg equilibrium which was important for genetic analysis. Non-representative cases might make a risk of outcome bias. Secondly, multiple genetic polymorphism investigations are necessary to realize precision administration. UGT1A4*3 may be one of the genetic polymorphism which can influence the concentration and therapeutic effect of LTG. Zhou *et al* [35] indicated that polymorphisms in HNF4α, ABCG2 and ABCB1 were associated with CDR of LTG. Therefore, much more single nucleotide polymorphisms involved in LTG pharmacokinetics can be further evaluated to provide factor when determine pharmacokinetic model for LTG dose adjustment. Finally, only three studies of Chinese reported the efficacy of LTG between different genotypes. It should be cautious with result of definitive correlation. Insufficient sample size in the meta-analysis might limit the reliability and accuracy.

In summary, current evidence indicated that UGT1A4*3 polymorphisms might influence CDR level and efficacy of LTG therapy. LTG administration is necessary to follow the principle of individualization.

## Supporting information

S1 Checklist PRISMA 2020 checklist. (DOCX)

S1 Table Detailed values of the outcomes in included studies and the values used to build graph after unifying. (DOCX)
