# Supplementary material for: UGT1A4*3 polymorphism influences serum concentration and therapeutic effect of lamotrigine for epilepsy treatment: A meta-analysis
Source: PLoS One. 2024 Jul 18;19(7):e0307377. doi: 10.1371/journal.pone.0307377 (PMC11257390; doi:10.1371/journal.pone.0307377)
Supplement: S1 Table — (DOCX) [file pone.0307377.s002.docx]

S1 Table. Detailed values of the outcomes in included studies and the values used to build graph after unifying

| **Author(year)** | **Outcomes** | | |
| --- | --- | --- | --- |
|  | **Mean serum concentration** | **CDR(μg/mL per mg/kg)** | **Efficacy** |
| Suzuki 2019[21] | TT(49):12.4±6.6μmol/L  TG(18):9.9±4.7μmol/L  **After unifying the unit to μg/ml:**  TT(49):3.17±1.69μg/ml  TG(18):2.53±1.2μg/ml |  |  |
| Petrenaite 2022[22] | TT(157):17.33±9.99μmol/L  TG(38):16.32±11.10μmol/L  **After unifying the unit to μg/ml:**  TT(157):4.44±2.56 μg/ml  TG(38):4.18±2.84 μg/ml |  |  |
| Gulcebi 2011[23] | TT(11):3.5±0.69mg/L  TG/GG (24)：2.4±1.05mg/L  **After unifying the unit to μg/ml:**  TT(11):3.5±0.69μg/ml  TG/GG (24):2.4±1.05μg/ml |  |  |
| Zhou 2015[24] | TT(64):2.61 μg/ml (2.00-3.92)  TG+GG(55):3.00 μg/ml(2.27-4.10)  **After using Min and Max data for estimated SD [19]:**  TT(64):2.61±0.48 μg/ml  TG+GG(55):3.00±0.46 μg/ml | TT:1.26(0.98-1.63)  TG+GG:1.35(1.00-1.48)  **After using Min and Max data for estimated SD[19]:**  TT(64):1.26±0.1625  TG+GG(55):1.35±0.12 |  |
| Liu 2015[25] | TT(34):2.01±0.75μg/ml  TG+GG(22):1.98±1.28μg/ml | TT(34): 0.87±0.35  TG+GG(22): 1.20±0.81 |  |
| Lou 2021[26] | TT(32):3.80 μg/ml (3.10-4.98)  TG(17):4.44 μg/ml (1.73-5.91)  **After using Min and Max data for estimated SD[19]:**  TT(32):3.80±0.47 μg/ml  TG(17):4.44±1.045 μg/ml | TT(32):0.96(0.64-1.59)  TG(17):0.92(0.55-1.71)  **After using Min and Max data for estimated SD[19]:**  TT(32):0.96±0.2375  TG(17):0.92±0.29 |  |
| Liu 2014[27] | TT(11):1.95±0.81 μg/ml  TG/GG(9):1.71±1.36μg/ml | TT(11):0.87±0.37  TG/GG(9):1.19±0.89 |  |
| Yang 2013[28 ] |  | TT(81):4.503±1.4701  TG+GG(22):2.325±0.5988 | TT(81): good65, poor16  TG+GG(22): good7, poor15 |
| Chang 2014[17] |  | TT:4.51±0.66 TG+GG:2.33±2.93 | TT(81): good64, poor17  TG+GG(22): good7, poor15 |
| Du 2016[29] |  | TT(74):2.77±0.87  TG(26):2.18±0.74  GG(2):1.95±0.49  **After the TG and GG group merged [20]**:  TG+GG(28):2.16±0.72 | TT(74): good56, poor18  TG+GG(28): good10, poor18 |
| Reimers 2016[30] |  | individuals heterozygous for UGT1A4*3 had a significantly lower CDR than individuals with wild-type (TT) without detailed data |  |

Molecular weight of LTG : 256.09
